# Predialysis nephrology care and dialysis-related health outcomes among older adults initiating dialysis

**DOI:** 10.1186/s12882-016-0324-5

**Published:** 2016-07-29

**Authors:** Michael J. Fischer, Kevin T. Stroupe, James S. Kaufman, Ann M. O’Hare, Margaret M. Browning, Min-Woong Sohn, Zhiping Huo, Denise M. Hynes

**Affiliations:** 1 Medicine/Nephrology, Jesse Brown VA Medical Center, University of Illinois Medical Center, Chicago, IL USA; 2 Center of Innovation for Complex Chronic Care, Edward Hines, Jr. VA Hospital, Hines, IL USA; 3 Department of Public Health Sciences, Loyola University Chicago, Maywood, IL USA; 4 VA Information Resource Center, Edward Hines, Jr. VA Hospital, Hines, IL USA; 5 Medicine/Nephrology, VA New York Harbor Healthcare System, New York, NY USA; 6 New York University School of Medicine, New York, NY USA; 7 Medicine/Nephrology, VA Puget Sound Healthcare System, Seattle, WA USA; 8 Medicine/Nephrology, Group Health Research Institute, University of Washington, Seattle, WA USA; 9 Department of Public Health Sciences, University of Virginia, Charlottesville, VA USA; 10 Medicine/Health Promotion Research, School of Public Health, University of Illinois at Chicago, Chicago, IL USA

**Keywords:** Dialysis, Elderly, Nephrology care

## Abstract

**Background:**

Predialysis nephrology care is associated with lower mortality and rates of hospitalization following chronic dialysis initiation. Whether more frequent predialysis nephrology care is associated with other favorable outcomes for older adults is not known.

**Methods:**

Retrospective cohort study of patients ≥66 years who initiated chronic dialysis in 2000–2001 and were eligible for VA and/or Medicare-covered services. Nephrology visits in VA and/or Medicare during the 12-month predialysis period were identified and classified by low intensity (<3 visits), moderate intensity (3–6 visits), and high intensity (>6 visits). Outcome measures included very low estimated glomerular filtration rate, severe anemia, use of peritoneal dialysis, and receipt of permanent vascular access at dialysis initiation and death and kidney transplantation within two years of initiation. Generalized linear models with propensity score weighting were used to examine the association between nephrology care and outcomes.

**Results:**

Among 58,014 patients, 46 % had none, 22 % had low, 13 % had moderate, and 19 % had high intensity predialysis nephrology care. Patients with a greater intensity of predialysis nephrology care had more favorable outcomes (all *p* < 0.001). In adjusted models, patients with high intensity predialysis nephrology care were less likely to have severe anemia (RR = 0.70, 99 % CI: 0.65–0.74) and more likely to have permanent vascular access (RR = 3.60, 99 % CI: 3.42–3.79) at dialysis initiation, and less likely to die within two years of dialysis initiation (RR = 0.80, 99 % CI: 0.77–0.82).

**Conclusion:**

In a large cohort of older adults treated with chronic dialysis, greater intensity of predialysis nephrology care was associated with more favorable outcomes.

**Electronic supplementary material:**

The online version of this article (doi:10.1186/s12882-016-0324-5) contains supplementary material, which is available to authorized users.

## Background

Several studies have demonstrated that absent, infrequent, or late nephrology care prior to dialysis initiation for patients with end-stage kidney disease (ESKD) is associated with significantly higher subsequent mortality and prolonged hospitalizations [1–4]. However, few of these studies included older patients [5–9], despite their high burden of ESKD treated with chronic dialysis. Compared with adults under the age of 60, incidence rates of treated ESKD are more than 2-fold higher in those aged 65–69 years and 3-fold higher in those aged 80–84 years [10].

Among prior studies that included older adults, neither the frequency of predialysis nephrology care obtained nor detailed information about a variety of clinical outcomes was reported [5, 6, 8]. The frequency of predialysis nephrology care visits may be especially important because decisions and interventions for dialysis planning and preparation often do not occur at a single point in time but rather as a process that unfolds over time [11, 12]. In a prior manuscript, we reported that older adults with frequent nephrology care (>6 visits) in the year prior to initiating dialysis had nearly half the hospital days and significantly lower total healthcare costs during the first year of chronic dialysis compared with those with less frequent or absent nephrology care [13].

Similarly, an examination of a broad range of outcomes beyond mortality is crucial for a proper understanding of the care for older patients with ESKD because they may value a range of outcomes beyond survival. Outcomes such as loss of independence and functional decline are relatively common after dialysis initiation among high risk populations of older adults [14, 15]. Moreover, since older adults have a higher burden of competing comorbid medical conditions, a more unpredictable course of kidney disease progression, and a higher risk of death than younger patients, it is unclear whether associations between predialysis nephrology care and more favorable outcomes hold true in this complex population [11, 16].

The objective of this present analysis was to evaluate the relationship between predialysis nephrology care and a range of dialysis-related clinical outcomes among older adult patients. Specifically, we examined the relationship between frequency of predialysis nephrology visits and outcomes at dialysis initiation (e.g., very low kidney function, severe anemia, receipt of peritoneal dialysis, use of permanent vascular access) and health outcomes after initiation (e.g., transplantation, mortality) among a large cohort of older adults with incident ESKD initiating dialysis.

## Methods

### Study design and sample

We conducted a retrospective cohort study of dialysis-related health outcomes at the time of dialysis initiation and during the two-year period afterwards among older adults who initiated chronic dialysis between January 1, 2000 and December 31, 2001 from our previously reported cohort [13, 17]. Medicare is the payer of most chronic dialysis care in the United States, while the Department of Veterans Affairs (VA) may provide such care to Veterans. Therefore, we included patients who were eligible for Medicare and/or VA-covered services during the 12-month period preceding dialysis initiation (i.e., predialysis period).

Dialysis initiation was identified using the United States Renal Data System (USRDS) national ESKD registry linked to Medicare claims and VA administrative data [18, 19]. To ensure that patients were eligible for Medicare-covered services throughout the 12-month predialysis period, we restricted our sample to patients who were ≥ 66 years old at dialysis initiation. To ensure adequate capture of healthcare utilization information, we excluded patients who (1) were enrolled in Medicare but did not have Medicare as their primary payer during this period, (2) were enrolled in Medicare managed care (i.e., Medicare Advantage) plans, or (3) had no healthcare use in either VA or Medicare during the predialysis period [7, 13, 17].

### Variables

#### Patient characteristics

We obtained data on patient characteristics (e.g., age, gender, race, ethnicity, body mass index) from Medicare enrollment files, the USRDS Patient and Medical Evidence Files, and VA administrative sources [18, 20, 21]. Co-morbidities were determined from diagnostic and procedure codes in Medicare claims data and national VA administrative data during the predialysis period [18, 20, 21]. To categorize socioeconomic status, we used zip-code-based median household income information from 2000 Census data [22]. To categorize access to care, we obtained county-level healthcare characteristics from the Area Resource File, including short-term hospital and physician density, and the urban/rural nature of a zip code of patient residence at dialysis initiation from the VA Planning Systems Support Group (PSSG) [23, 24]. Potential geographic variation in the intensity of predialysis care was categorized using census region [22].

The study cohort included Veteran and non-Veteran patients. Veterans were defined as individuals who used VA healthcare services, were enrolled in the Veterans Health Administration, or received pension or compensation from VA [13, 17]. All other study patients were classified as “non-Veterans”, which may include Veterans who have not received health care or benefits from VA [13, 17].

#### Nephrology care

Episodes of outpatient nephrology care as well as other utilization (e.g., primary care, hospitalizations) during the predialysis period were identified using both the Medicare Carrier files and VA administrative data as described previously [13, 17]. Nephrology care was defined as the presence of any of the following during the predialysis period: nephrology clinic visit (VA), outpatient hypertension clinic visit with a nephrology provider (VA), and nephrology provider visit (Medicare). Because visits coded as level 1 in Medicare do not require interaction with a nephrology practitioner, they were excluded. Participants were categorized into four mutually exclusive groups by intensity of predialysis nephrology care: no nephrology care, low intensity (1–3 visits), moderate intensity (4–6 visits) and high intensity predialysis nephrology care (>6 visits) in the year prior to dialysis initiation [5–7, 13, 17]. We also examined intensity of predialysis nephrology care among the subgroup referred late to a nephrologist, which was defined as having the first visit to a nephrologist less than 3 months before dialysis initiation [4, 5, 13, 17, 25–27], in order to also account for the effect of timing of predialysis nephrology care on outcomes.

#### Outcomes at dialysis initiation

We assessed the following intermediate clinical outcomes at the time of dialysis initiation as reported in the USRDS Medical Evidence file based on current guidelines and/or definitions from prior clinical studies: 1) whether a patient had very low estimated glomerular filtration rate (eGFR), defined as an eGFR ≤5 mL/min/1.73 m^2^ and 2) whether a patient had severe anemia, defined as a hemoglobin level <9 g/dL [28].

We assessed the following process measures at dialysis initiation: 1) whether a patient had received peritoneal dialysis within 60 days of dialysis initiation using the USRDS Treatment History files and VA databases (for patients receiving dialysis within VA or VA fee basis) and 2) whether a patient had received permanent vascular access placement for dialysis during the 2 years before dialysis initiation, based on the presence of at least one Current Procedural Terminology (CPT) and International Classification of Disease – 9^th^ Revision (ICD-9) procedural code using Medicare claims and VA administrative data. Permanent vascular access was classified as arteriovenous fistula (fistula), arteriovenous graft (graft), and other (fistula or graft) using CPT and ICD-9 codes. Because patients intending to receive peritoneal dialysis do not require permanent vascular access placement, we excluded patients who received peritoneal dialysis within 60 days of dialysis initiation from our analyses of permanent vascular access placement.

#### Outcomes following dialysis initiation

Outcomes after dialysis initiation included: 1) death within 2 years of dialysis initiation, which was ascertained from the USRDS Patients’ file [18] and VA Beneficiary Identification Record Locator Subsystem death file, and 2) kidney transplant within 2 years of dialysis initiation, which was determined from the USRDS Patients’ files.

### Statistical methods

We compared characteristics of all participants and outcomes by intensity of predialysis nephrology care using ANOVA or Chi-square tests. Using the same statistical tests, we also compared outcomes across intensity of predialysis nephrology care in the subgroup with late nephrology care (first nephrology visit <3 months before dialysis initiation) and after stratification by age and burden of comorbidity. Additionally, we computed the number of days from dialysis initiation to death and used Kaplan-Meier estimates to display survival by intensity of predialysis nephrology care, and the log-rank test was used to determine significant comparisons.

We used generalized linear models (GLM) with robust variance estimates to further examine the association of intensity of predialysis nephrology care with outcomes (described above) [29, 30]. We used a Poisson distribution with a log link function to calculate risk ratios (RR), which facilitates interpretation of results since there is an increasing differential between relative risk ratios and odds ratios as the incidence of an outcome increases. In order to account for potential biases from non-random patient assignment into intensity of predialysis nephrology care group, we used inverse probability weighting with the weights calculated based on propensity score of patients being placed in each of the predialysis nephrology care intensity groups [19]. Propensity scores were computed from 3 logistic models that predicted the probability of membership in 1 of the 3 groups rather than the no predialysis care group, adjusting for predialysis patient characteristics (i.e., demographics, comorbidities, healthcare system used) and characteristics of the patient’s geographic location. The weights were defined as one over the propensity score for patients in each predialysis intensity group and one over one minus the propensity score for patients with no predialysis nephrology care [19].

All analyses were conducted using STATA/MP version 14.0 [31]. In order to account for multiple tested outcomes, a *p*-value < 0.01 was considered significant for these analyses.

## Results

### Participant characteristics

After exclusions, the final analytic cohort comprised 58,014 patients (Additional file 1: Figure S1). Overall, 46 % had no predialysis nephrology care, 22 % had low intensity care, 13 % had moderate intensity care, and 19 % had high intensity care. With increasing intensity of predialysis nephrology care, patients were younger and more likely to be male (*p* < 0.001) (Table 1). Some chronic co-morbidities (e.g., myocardial infarction, congestive heart failure) were less common in patients with greater intensity of predialysis nephrology care, while others were more common (e.g., diabetes mellitus, hypertension) (*p* < 0.001). As intensity of predialysis nephrology care increased, the proportion of patients living in urban residence increased and the proportion with median income < $30,000 decreased (*p* < 0.001). Sensitivity analyses (Additional file 2: Table S1), which collapsed groups into absent or present nephrology care, confirm that propensity weighting eliminated significant inter-group differences.

**Table 1:** Patient characteristics by intensity of predialysis nephrology care

Characteristic	Overall	No visits	Low intensity^a^	Moderate intensity^a^	High intensity^a^	*P*-value
	*N* = 58,014	*N* = 26,798	*N* = 12,566	*N* = 7,690	*N* = 10,960	
Patient Characteristics						
Age (yrs)^b^	75.7	76.1	75.5	75.2	75.2	<0.001
Female, %	50.1	52.2	50.7	48.0	46.1	<0.001
*Race*, %						0.16
White	75.2	75.3	75.4	74.9	74.9	
African American	20.9	20.7	20.7	21.7	21.1	
Other	3.9	4.0	3.9	3.4	4.0	
Hispanic Ethnicity, %	9.0	9.2	9.6	8.7	8.3	0.002
Body Mass Index (kg/m^2^)^b^	26.0	25.8	25.9	26.4	26.3	<0.001
Geographic Characteristics						
Hospital Density^c^	20.0	20.0	21.3	20.6	18.2	<0.001
Physician Density^d^	2.2	2.3	2.2	2.1	2.2	<0.001
Urban Residence	84.1	84.5	82.5	82.7	85.9	<0.001
Median Income < $30,000^e^	23.1	23.6	24.0	23.3	20.7	<0.001
*Region*, %						<0.001
Northeast	14.5	14.4	14.2	14.4	15.1	
Midwest	25.7	28.6	24.5	23.8	21.4	
South	38.1	33.5	39.7	41.0	45.2	
West	20.3	22.2	20.0	19.1	17.1	
Predialysis Healthcare Use						
Primary Care Visits^b^	4.2	4.1	4.5	4.5	4.0	<0.001
Inpatient Days^b^	12.8	14.8	14.5	9.9	7.7	<0.001
Inpatient Admissions^b^	1.5	1.5	1.6	1.3	1.2	<0.001
Predialysis Comorbidities, %						
Myocardial Infarction	10.8	13.3	10.7	8.0	6.8	<0.001
Congestive Heart Failure	57.6	62.1	59.7	53.0	47.2	<0.001
Cerebrovascular Disease	11.5	12.5	12.4	10.0	9.3	<0.001
Chronic Obstructive Pulmonary Disease	25.9	29.0	26.3	22.6	20.1	<0.001
Diabetes mellitus	52.4	50.6	54.4	55.9	52.2	<0.001
Hypertension	92.8	88.7	95.2	96.9	97.5	<0.001
Peripheral vascular disease	22.0	23.0	23.7	21.3	18.2	<0.001
Veteran, %	12.8	10.1	14.0	16.1	15.6	<0.001

^a^Patients were categorized by intensity of predialysis nephrology care: low intensity care (1–3 nephrology visits), moderate intensity care (4–6 nephrology visits), and high intensity care (>6 nephrology visits)

^b^Mean value

^c^Number of hospitals per million persons (1990)

^d^Number of physicians per thousand persons (1990)

^e^1999 data (US dollars)

### Dialysis-related outcomes by intensity of predialysis nephrology care

Patients who received a greater intensity of predialysis nephrology care had a higher prevalence of permanent vascular access (both fistula and graft) and a lower prevalence of severe anemia and very low eGFR at the time of dialysis initiation (Table 2). Similarly, use of peritoneal dialysis within 60 days of dialysis initiation was more frequent in patients with greater intensity of predialysis nephrology care (*p* < 0.001). The percentage of patients who died within 2 years of dialysis initiation was 59.7 % (15,991/26,789), 55.0 % (6,916/12,566), 48.0 % (3,694/7,690), and 42.7 % (4,689/10,960) among those who received no, low intensity, moderate, or high intensity predialysis nephrology care, respectively (*p* < 0.001). The percentage of patients who received a kidney transplant within 2 years of dialysis initiation was 0.4 % (106/26,798), 0.6 % (76/12,566), 1.0 % (75/7,690), and 1.5 % (164/10,960) among patients who received no, low intensity, moderate, or high intensity predialysis nephrology care, respectively, (*p* < 0.001). Upon analyses stratified by sex and race, outcomes by intensity of predialysis nephrology care were not significantly different and consistent across these subgroups.

**Table 2:** Dialysis-related health outcomes by intensity of predialysis nephrology care

Characteristic	No visits	Low intensity^a^	Moderate intensity^a^	High intensity^a^	*P*-value
	*N* = 26,798	*N* = 12,566	*N* = 7,690	*N* = 10,960	
At Dialysis Initiation, %					
Very low eGFR^c^	10.1	7.3	6.9	7.0	<0.001
Severe anemia^d^	28.5	27.7	24.3	19.4	<0.001
Permanent vascular access^b^	13.7	22.6	38.7	54.1	<0.001
Fistula	7.8	13.6	23.0	33.9	<0.001
Graft	3.7	6.5	13.5	17.7	<0.001
Other (graft or fistula)	2.2	2.6	2.6	3.2	<0.001
Peritoneal Dialysis	4.9	7.3	10.3	11.4	<0.001
At Dialysis Follow-up, %					
Mortality at 2 years	59.7	55.0	48.0	42.7	<0.001
Kidney transplant at 2 years	0.4	0.6	1.0	1.5	<0.001

^a^Patients were categorized by intensity of predialysis nephrology care: low intensity care (1–3 nephrology visits), moderate intensity care (4–6 nephrology visits), and high intensity care (>6 nephrology visits)

^b^ *N* = No Visits = 25,490; Low Intensity = 11,649; Moderate Intensity = 6,896; High Intensity = 9,708

^c^eGFR ≤5 mL/min/1.73 m^2^

^d^Hemoglobin level <9 g/dL

**Fig. 1:**
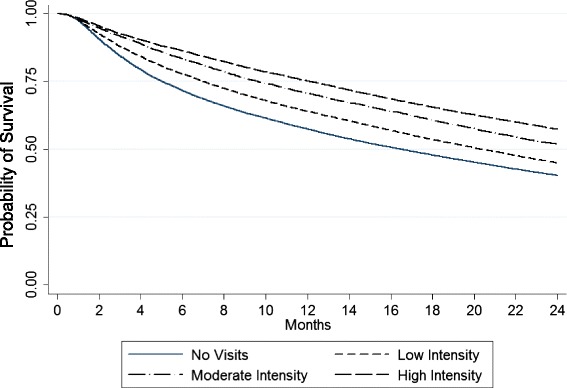
Patient survival by intensity of predialysis nephrology care

Survival was also longer among patients with a greater intensity of predialysis nephrology care (Fig. 1) (*p* < 0.001). Following dialysis initiation, median days of survival were 502, 618, 730, and 730 among patients who received no, low intensity, moderate, or high intensity nephrology care in the predialysis period, respectively, (*p* < 0.001).

Results were similar in subgroup analyses among patients with late nephrology care (first nephrology visit <3 months before dialysis initiation) (Table 3), among patients ≥ 75 years, among those with three or more comorbid conditions (e.g., diabetes + hypertension + cardiovascular disease), and among those with differing levels of eGFR at dialysis initiation (data not shown).

**Table 3:** Dialysis-related health outcomes by intensity of predialysis nephrology care among patients with late predialysis nephrology referral

Characteristic	Low intensity^a^	Moderate intensity^a^	High intensity^a^	*P*-value
	*N* = 6,962	*N* = 1,093	*N* = 359	
At Dialysis Initiation, %				
Very low eGFR^c^	6.6	4.8	7.5	0.04
Severe anemia^d^	27.0	23.2	20.1	0.001
Permanent vascular access^b^	21.7	39.4	41.3	<0.001
Fistula	12.8	18.8	19.5	<0.001
Graft	6.5	17.7	19.5	<0.001
Other (graft or fistula)	2.4	3.1	2.3	0.46
Peritoneal Dialysis	7.9	11.4	15.6	<0.001
At Dialysis Follow-up, %				
Mortality at 2 years	55.1	48.0	45.7	<0.001
Kidney transplant at 2 years	0.6	0.6	0.3	0.72

^a^Patients were categorized by intensity of predialysis nephrology care: low intensity care (1–3 nephrology visits), moderate intensity care (4–6 nephrology visits), and high intensity care (>6 nephrology visits)

^b^ *N* = Low Intensity = 6,409; Moderate Intensity = 968; High Intensity = 303

^c^eGFR ≤5 mL/min/1.73 m^2^

^d^Hemoglobin level <9 g/dL

### Association intensity of predialysis nephrology care with dialysis-related outcomes

Significant independent associations were observed between intensity of predialysis nephrology care and dialysis-related outcomes (Table 4). In particular, compared with the referent group (i.e., no predialysis nephrology care), the prevalence of severe anemia at the time of dialysis initiation was 4 % lower for patients with low intensity predialysis nephrology care (RR = 0.96, 99 % CI: 0.92 to 1.01), 16 % lower for patients with moderate intensity predialysis nephrology care (RR = 0.84, 99 % CI: 0.79 to 0.90) and 30 % lower for patients with high intensity predialysis nephrology care (RR = 0.70, 99 % CI: 0.65 to 0.74). The risk of permanent vascular access at dialysis initiation was 57 % greater with low intensity (RR = 1.57, 99 % CI: 1.48 to 1.67), 161 % greater with moderate intensity (RR = 2.61, 99 % CI: 2.45 to 2.77), and 260 % greater with high intensity predialysis nephrology care (RR = 3.60, 99 % CI: 3.42 to 3.794). The risk of death within 2 years after dialysis initiation was 6 % lower among patients with low intensity (RR = 0.94, 99 % CI: 0.92 to 0.97), 13 % lower for patients with moderate intensity (RR = 0.87, 99 % CI: 0.84 to 0.91), and 20 % lower for patients with high intensity predialysis nephrology care (RR = 0.80, 99 % CI: 0.77 to 0.82).

**Table 4:** Association of dialysis-related health outcomes with Intensity of predialysis nephrology care

Predialysis nephrology care	Adjusted relative risk ratio (99 % CI)^a^		
	Low intensity^b^	Moderate intensity^b^	High intensity^b^
	*N* = 12,566	*N* = 7,688	*N* = 10,956
At Dialysis Initiation, %			
Very low eGFR^c^	0.72 (0.66 to 0.80)	0.67 (0.59 to 0.77)	0.66 (0.59 to 0.75)
Severe anemia^d^	0.96 (0.92 to 1.01)	0.84 (0.79 to 0.90)	0.70 (0.65 to 0.74)
Permanent vascular access^b^	1.57 (1.48 to 1.67)	2.61 (2.45 to 2.77)	3.60 (3.42 to 3.79)
Fistula	1.66 (1.52 to 1.80)	2.72 (2.50 to 2.97)	3.85 (3.58 to 4.14)
Graft	1.65 (1.46 to 1.87)	3.20 (2.84 to 3.61)	4.28 (3.83 to 4.77)
Other (graft or fistula)	1.15 (0.96 to 1.38)	1.18 (0.94 to 1.49)	1.60 (1.30 to 1.97)
Peritoneal Dialysis	1.44 (1.29 to 1.60)	2.01 (1.77 to 2.27)	2.12 (1.90 to 2.37)
At Dialysis Follow-up, %			
Mortality at 2 years	0.94 (0.92 to 0.97)	0.87 (0.84 to 0.91)	0.80 (0.77 to 0.82)
Kidney transplant at 2 years	1.41 (0.95 to 2.10)	2.13 (1.39 to 3.27)	2.72 (1.91 to 3.88)

^a^Weighted by propensity scores derived from logistic regression models including age, sex, race, Hispanic ethnicity, body mass index, hospital and physician density, urban residence, median income, region, predialysis comorbidities (listed in Table 1), veteran status, and predialysis outpatient care venue. No predialysis nephrology care visits is the reference group in each of the 3 propensity score models

^b^Patients were categorized by intensity of predialysis nephrology care: low intensity care (1–3 nephrology visits), moderate intensity care (4–6 nephrology visits), and high intensity care (>6 nephrology visits). Patients with no predialysis nephrology care were the reference group

^c^eGFR <5 mL/min/1.73m2

^d^hemoglobin level <9 g/dL

^e^ *N* = Low Intensity = 11,649; Moderate Intensity = 6,894; High Intensity = 9,708

## Discussion

In older adults treated with chronic dialysis, greater intensity of predialysis nephrology care was associated with more favorable health parameters and outcomes at the time of dialysis initiation and for the first two years following initiation. A greater number of predialysis visits were independently associated with a lesser likelihood of having a very low eGFR and severe anemia and a greater risk of permanent vascular access and use of peritoneal dialysis at dialysis initiation. Moreover, a higher number of predialysis visits was associated with decreased risk of death and higher chance of kidney transplantation during follow up. Results were consistent in subgroup analyses among very older adults, those with a substantial burden of comorbidity, and those whose initial visit occurred <3 months before dialysis initiation.

In contrast to most prior studies of predialysis nephrology care, which focused only on mortality after dialysis initiation, we evaluated outcomes at dialysis initiation (e.g., placement of permanent vascular access, presence of severe anemia, use of peritoneal dialysis) in older patients, finding that nearly all were more favorable with more frequent predialysis nephrology care. These results raise the question of whether improving the frequency of predialysis care for older patients with kidney disease represents an opportunity to improve preparation, treatment of complications, and modality selection for chronic dialysis. Cohort studies in the United States and Europe have reported that >50 % of incident older dialysis patients begin dialysis with a catheter instead of arteriovenous graft or fistula (i.e., permanent vascular access), and that catheter use is associated with up to a 70 % increase in death at 1-year among these older dialysis patients [32, 33]. Comparable to our results, Avorn et al. found that more frequent predialysis nephrology care (≥3 visits) was independently associated with a 1.5 fold increase in permanent vascular access in a mixed Medicaid and Medicare cohort [8]. Similarly, many older Medicare recipients initiate dialysis with a hemoglobin <9 g/dL, despite current guideline recommendations [34]. Severe anemia, as defined in this study by a hemoglobin < 9 g/dL, has also been observed to be independently associated with the additional burden of transfusions [35]. Peritoneal dialysis appears to be underutilized in older adults, despite the observation that many older patients do not cope well with in-center hemodialysis [36]. A recent literature review concluded that most older patients have the requisite physical and cognitive skills to successfully perform peritoneal dialysis, and have excellent compliance and success with this modality [37]. In addition to similar survival compared with similar aged individuals on hemodialysis, older patients treated with peritoneal dialysis may have better quality of life [37, 38].

We are not aware of prior studies reporting the relationship between frequency of predialysis nephrology and access to kidney transplantation in older adults. Although overall rates of kidney transplantation were low, we did observe higher rates of kidney transplantation in patients who received more intensive nephrology care. While kidney transplantation is less common in older compared with younger patients with ESKD, a steady increase in kidney transplantation in older adults has been observed during the last decade [39, 40]. Similar to their younger counterparts, older patients who undergo transplantation have lower mortality rates and higher quality of life compared with those receiving chronic dialysis [39, 40].

Our results reporting lower 2-year mortality among older patients who received more frequent predialysis nephrology care are broadly consistent with prior studies. In three large cohort studies of older Medicare recipients, infrequent (<5 visits before dialysis initiation) or late (<3 months of care before dialysis initiation) nephrology care was independently associated with up to a 36 % increase in 1-year mortality [5, 6, 9]. In a recent examination of secular trends in timing of nephrology care for older Medicare patients, a large increase in timely nephrology referral prior to dialysis was observed, and referral to a nephrologist was associated with lower mortality. Although there was only a very modest improvement in patient survival over this time period, more timely referral to a nephrologist appeared to account for about half of this improvement [9, 41]. In a German study comparing 1-year mortality in adults with pre-ESKD who were > =75 years with those <75 years, late nephrology referral (<8 weeks before starting dialysis) was similarly associated with an increased risk of mortality in both older and young adults [32].

We are not advocating broad nephrology referral for all older patients with severe CKD. Decisions regarding implementing guideline recommendations and dialysis preparations for older patients are often particularly complex and challenging because of the burden of disability and functional compromise [42–45]. Although members of this cohort who received more frequent predialysis nephrology care experienced more favorable outcomes, it is important to note that our study was restricted to those who initiated chronic dialysis. Since many older patients have slow progressive loss of kidney function and die before progressing to dialysis initiation [46], predicting prospectively which ones will start chronic dialysis among large populations of older adults with severe CKD can be challenging and balancing the risks and benefits of relevant management strategies (e.g., permanent vascular access surgery) may not be straightforward [47]. A recent small study testing clinical vignettes of older patients with severe CKD among healthcare providers noted that providers would only refer 50 % of these patients to a nephrologist [48]. Both physician specialty (e.g., internist, geriatrician) and patient characteristics such as comorbidity burden, cognitive decline, and functional impairment were noted to influence referral decisions [48]. While we did not have data regarding important clinical attributes such as frailty and dementia, we did observe an association of improved dialysis-related outcomes with predialysis nephrology care even in patients with the highest degree of comorbid disease burden and advanced age. Clearly, a nuanced patient-centered approach is needed for decisions regarding nephrology referral for older adults with severe CKD.

There are limitations to this study. First, selection bias could impact our findings because predialysis nephrology care was allocated in a nonrandom manner and some characteristics differed between these groups. Patients with more rapid loss of kidney function might be both more ill and less likely to see a nephrologist. However, we included a robust number of important covariates that could have potentially confounded our results and employed a weighted propensity score approach to minimize this concern [19]. Second, lead time bias could affect our findings because patients with higher intensity nephrology care initiated dialysis at higher eGFR values, and may possibly have less severe disease. However, the relationship between intensity of predialysis nephrology care and outcomes was unchanged across strata of eGFR. Third, although the time period of this study and its data are from 2000 to 2002 and may not reflect current rates of nephrology referral for older adults, nephrology referral guidelines for patients with severe CKD have not changed since the conduct of this study; therefore, these findings remain relevant. Fourth, because all participants in this cohort initiated dialysis, we cannot comment on the impact of predialysis nephrology care on outcomes in older patients with severe CKD who did not reach ESKD because of death or less progressive CKD. Fifth, the lack of outpatient predialysis nephrology care may reflect the patient’s preference not to receive nephrology care, the provider’s preference to not refer certain patients for such care, or the its substitution for inpatient nephrology care, which was not captured in our data. Finally, the use of surgical codes to identify vascular access may not indicate that the access is actually being used for dialysis and as such, may overestimate its use. While the USRDS Medical Evidence File contains data regarding vascular access at dialysis initiation, it only began including this information in 2005 and hence this source could not be used for our cohort.

## Conclusions

In conclusion, more frequent predialysis nephrology care among older patients initiating chronic dialysis was associated with improved control of disease complications, preparation for dialysis, and patient survival. These findings suggest that in older patients expected to initiate chronic dialysis, more frequent nephrology care beforehand may translate into more favorable outcomes at the time of and within two years following dialysis initiation.

## Abbreviations

CI, confidence interval; CKD, chronic kidney disease; CPT, current procedural terminology; eGFR, estimated glomerular filtration rate; ESKD, end-stage kidney disease; Fistula, arteriovenous fistula; GLM, generalized linear models; Graft, arteriovenous graft; HMO, health maintenance organizations; ICD-9, international classification of disease – 9th revision; PSSG, planning systems support group; RR, relative risk; USRDS, United States renal data system; VA, veterans affairs

## Additional files

Additional file 1: Figure S1. Study Sample Selection. Selection of Study Sample. Description: flow diagram of data used to determine final analytic study cohort. (DOCX 67 kb) Additional file 2: Table S1. Sample Characteristics by Propensity Weighting. Patient Characteristics by Intensity of Predialysis Nephrology Care: Unweighted and Propensity Score Weighted Samples. Description: baseline characteristics of the unweighted study sample and propensity weighted sample. (DOCX 75 kb)
