# Supplementary material for: Predialysis nephrology care and dialysis-related health outcomes among older adults initiating dialysis
Source: BMC Nephrol. 2016 Jul 29;17:103. doi: 10.1186/s12882-016-0324-5 (PMC4966864; doi:10.1186/s12882-016-0324-5)
Supplement: Additional file 1: Figure S1. — Study Sample Selection. Selection of Study Sample. Description: flow diagram of data used to determine final analytic study cohort. (DOCX 67 kb) [file 12882_2016_324_MOESM1_ESM.docx]

**Supplementary Figure 1: Selection of Study Sample**

90,406 unique patients

whose first ESRD service date was in 2000 or 2001 and whose age was >66 at the first ESRD service (USRDS Core CD)

Excluded (n=27,756)

- Did not have any healthcare use in VA or Medicare in Predialysis period: 19,947
- Enrolled in Medicare Managed Care in Predialysis period: 15,144
- Did not have Medicare as primary payer for Medicare-covered services: 19,385

62,650 unique patients

Excluded (n=4,636)

- Missing Hemoglobin value: 3,904
- Missing eGFR information: 1,470

58,014 unique patients
