# Supplementary material for: Predialysis nephrology care and dialysis-related health outcomes among older adults initiating dialysis
Source: BMC Nephrol. 2016 Jul 29;17:103. doi: 10.1186/s12882-016-0324-5 (PMC4966864; doi:10.1186/s12882-016-0324-5)
Supplement: Additional file 2: Table S1. — Sample Characteristics by Propensity Weighting. Patient Characteristics by Intensity of Predialysis Nephrology Care: Unweighted and Propensity Score Weighted Samples. Description: baseline characteristics of the unweighted study sample and propensity weighted sample. (DOCX 75 kb) [file 12882_2016_324_MOESM2_ESM.docx]

**Supplementary Table 1. Patient Characteristics by Intensity of Predialysis Nephrology Care: Unweighted and Propensity Score Weighted Samples**

| **Characteristic** | | **Unweighted** | | | | | **Weighted** | | | |
| --- | --- | --- | --- | --- | --- | --- | --- | --- | --- | --- |
|  | | **No Visits**  **N=26,781** | | **Visitsa**  **N=31,212** | | **P-Value** | **No Visits** | **Visitsa** | **P-Value** | |
| **Patient Characteristics** | |  | |  | |  |  |  |  | |
| Age >75 | | 55.9 | | 51.1 | | <0.001 | 53.3 | 53.4 | 0.765 | |
| Female, % | | 52.2 | | 48.4 | | <0.001 | 50.0 | 50.0 | 0.959 | |
| *Race*, % | |  | |  | | 0.195 |  |  | 0.971 | |
| White | | 75.3 | | 75.1 | |  | 75.4 | 75.5 |  | |
| African American | | 21.1 | | 21.1 | |  | 20.7 | 20.7 |  | |
| Other | | 4.0 | | 3.8 | |  | 3.9 | 3.9 |  | |
| Hispanic Ethnicity, % | | 9.2 | | 8.9 | | 0.286 | 9.0 | 9.0 | 0.949 | |
| Body Mass Index (kg/m2)b | | 25.8 | | 26.2 | | <0.001 | 26.0 | 26.0 | 0.759 | |
| **Geographic Characteristics** | |  | |  | |  |  |  |  | |
| Hospital Density^c^ | | 20.0 | | 20.0 | | 0.912 | 20.1 | 20.1 | 0.984 | |
| Physician Density^d^ | | 2.3 | | 2.2 | | <0.001 | 2.2 | 2.2 | 0.863 | |
| Urban Residence | | 84.5 | | 83.8 | | 0.013 | 84.0 | 84.0 | 0.947 | |
| Median Income <$30,000^e^ | | 23.6 | | 22.7 | | 0.007 | 23.1 | 23.0 | 0.844 | |
| *Region*, % | |  | |  | | <0.001 |  |  | 0.994 | |
| Northeast | | 14.4 | | 14.6 | |  | 14.5 | 14.6 |  | |
| Midwest | | 28.6 | | 23.3 | |  | 25.8 | 25.9 |  | |
| South | | 33.5 | | 42.0 | |  | 38.1 | 37.9 |  | |
| West | | 22.2 | | 18.8 | |  | 20.3 | 20.3 |  | |
| Other | | 1.3 | | 1.5 | |  | 1.4 | 1.4 |  | |
| **Predialysis Comorbidities, %** |  | |  | | |  |  |  |  |  |
| Myocardial Infarction | 13.3 | | 8.7 | | | <0.001 | 10.9 | 10.8 | 0.960 |  |
| Congestive Heart Failure | 62.1 | | 53.7 | | | <0.001 | 57.8 | 57.8 | 0.976 |  |
| Cerebrovascular Disease | 12.4 | | 10.7 | | | <0.001 | 11.6 | 11.6 | 0.896 |  |
| Chronic Obstructive Pulmonary Disease | 29.0 | | 23.2 | | | <0.001 | 26.1 | 26.2 | 0.937 |  |
| Diabetes mellitus | 50.6 | | 54.0 | | | <0.001 | 52.5 | 52.6 | 0.876 |  |
| Hypertension | 88.7 | | 96.4 | | <0.001 | | 92.8 | 92.8 | 0.742 |  |
| Peripheral vascular disease | 23.0 | | 21.2 | | <0.001 | | 22.2 | 22.1 | 0.833 |  |

a includes low intensity, moderate intensity, and high intensity

b mean value

c number of hospitals per million persons (1990)

d number of physicians per thousand persons (1990)

e 1999 data (US dollars)
